# Molecular evidence supports the expansion of visceral leishmaniasis towards non-program districts of Nepal

**DOI:** 10.1186/s12879-019-4083-3

**Published:** 2019-05-21

**Authors:** Mitesh Shrestha, Medha Khatri-Chhetri, Ram Chandra Poudel, Jyoti Maharjan, Shyam Prakash Dumre, Krishna Das Manandhar, Basu Dev Pandey, Sher Bahadur Pun, Kishor Pandey

**Affiliations:** 1 Molecular Biotechnology Unit, Nepal Academy of Science and Technology (NAST), Khumaltar, GPO box: 3323, Lalitpur, Nepal; 2 Department of Immunogenetics, Institute of Tropical Medicine, Nagasaki University, Nagasaki, Japan; 3 Central Department of Biotechnology, Tribhuvan University, Kathmandu, Nepal; 4 Sukraraj Tropical and Infectious Disease Hospital, Kathmandu, Nepal

**Keywords:** Non-program districts, Visceral leishmaniasis, High altitude, Nepal

## Abstract

**Background:**

Visceral Leishmaniasis (VL) is caused by a protozoan parasite *Leishmania donovani* that is transmitted to humans by an infected female sandfly, *Phlebotomus argentipes*. VL is common in the Indian sub-continent including Nepal and efforts for its elimination are ongoing. However, expansion of disease towards the higher altitude areas, previously considered as VL free in Nepal, may impact the ability to achieve the elimination target by 2020.

**Methods:**

This was an exploratory study, where VL suspected patients living exclusively in the non-program districts of Nepal and presenting with fever > 2 weeks and splenomegaly was included. The patients’ blood samples were collected, and DNA was extracted. DNA was subjected to PCR amplification and subsequent sequencing. Additionally, past 10 years data of VL cases from the national databases were analysed to see the trends of the disease in program and non program districts.

**Results:**

Analysis of the past 10 years data revealed that trend of VL cases significantly decreased in the program districts (*p* = 0.001) while it increased in the non-program districts (*p* = 0.002). The national trend for overall incidence of VL also significantly decreased over this time period. Limited number of patients’ samples (*n* = 14) were subjected to molecular investigation, and four patients were found to be positive for *Leishmania* species by PCR. Interestingly, these cases in non-program districts were indeed also *L. donovoni complex*. All four patients were male with age ranges from 10 to 68 years. GenBank BLAST of the obtained DNA sequences confirmed identified specimens as *L. donovani complex*. We identified additional VL cases from non-program districts (including the high lands) of Nepal, indicating that the infection could be an emerging threat for the non-program areas of Nepal.

**Conclusion:**

The demonstration of VL cases in areas initially considered non-endemic has raised concern about on-going transmission in those regions and may trigger subsequent government plan and action to include those areas in the elimination program. Thus, the government should consider revising the disease control programs to accommodate non-program districts for achieving the VL elimination goal set for 2020.

## Background

Leishmaniasis, one of the neglected tropical diseases, is caused by a protozoan parasite, *Leishmania* spp. and transmitted by bite of infected female sandfly (*Phlebotomus* spp. and *Lutzomyia* spp.) [1]. Globally, 350 million people from over 98 countries in the tropical and sub-tropical regions are at risk of this disease [2]. Leishmaniasis is categorized as cutaneous, muco-cutaneous and visceral leishmaniasis (VL) based on the parasite species specific clinical manifestations [3, 4]. VL is caused by *Leishmania donovani* and is the most common clinical presentation in the Indian sub-continent including Nepal [5, 6]. Continuous high grade fever, weight-loss and an enlarged liver and spleen are the most common features of VL patients, and if not treated, case fatality rate may reach as high as 95%, though it can be reduced to < 3% with proper clinical management on time [7]. The disease is now expanding to the newer areas due to rapid urbanization, sharp increase in migration and adaptation of the *Leishmania* parasite to additional vectors and mammalian hosts [8].

VL has been identified by WHO as a public health problem and target for its elimination (< 1 case/10,000 people/year) from the Indian sub-continent including Nepal has been set for 2020 [9, 10]. As of now, VL is thought to be endemic in 12 districts of the low land Terai region of Nepal. Therefore these 12 districts have been considered as program districts and attain higher priorities for public health support programs in Nepal while the remaining 63 districts are regarded as non-program districts [9, 10]. The recent surge in the number of cases reported from non-program districts has become a serious issue in achieving VL elimination from this region [11–14]. The main aim of the present study is identification and confirmation of *Leishmania* spp. by employing a molecular approach in VL suspected patients’ samples from non-program districts of Nepal.

## Methods

### Study design, enrolment and sample collection

**Fig. 1:**
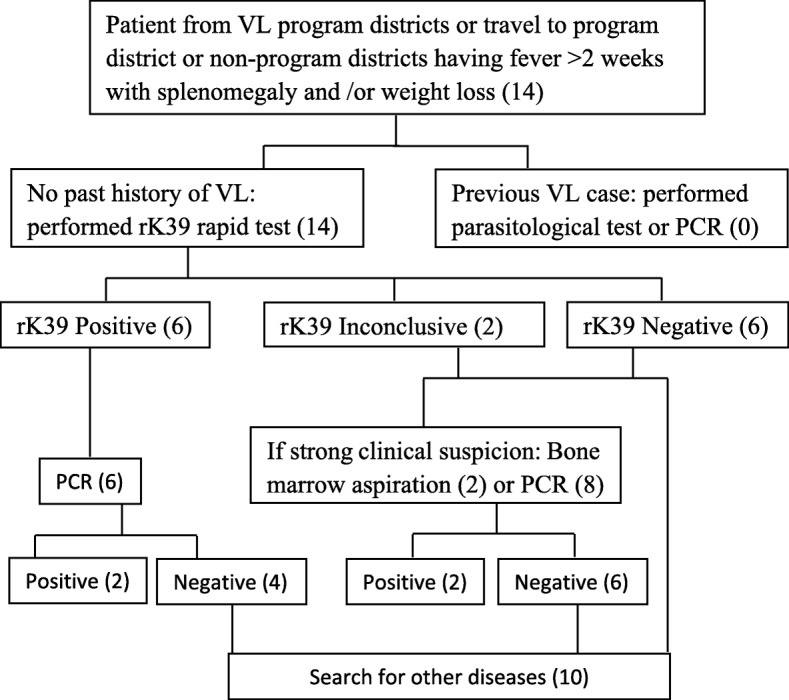
Visceral leishmaniasis diagnostic algorithm in Nepal, inside the bracket shows number of cases

This was an exploratory study conducted at Sukraraj Tropical and Infectious Disease Hospital (STIDH), Kathmandu, which serves as a central referral hospital for diagnosis and treatment of infectious and tropical diseases in Nepal. STIDH receives VL patients from both program and non- program districts throughout the country. Suspected VL patients were defined as patients with history of fever for more than two weeks, weight loss, splenomegaly, hepatomegaly, and sign of anemia, according to the National guidelines [10]. Those who met clinical case definition according to national guidelines were tested rK39 dipstick test. Bone marrow aspiration was performed for confirmation when a patient had a previous history of VL or a history of travel or living in known VL endemic areas with negative rK39 result (Fig. 1).

**Fig. 2:**
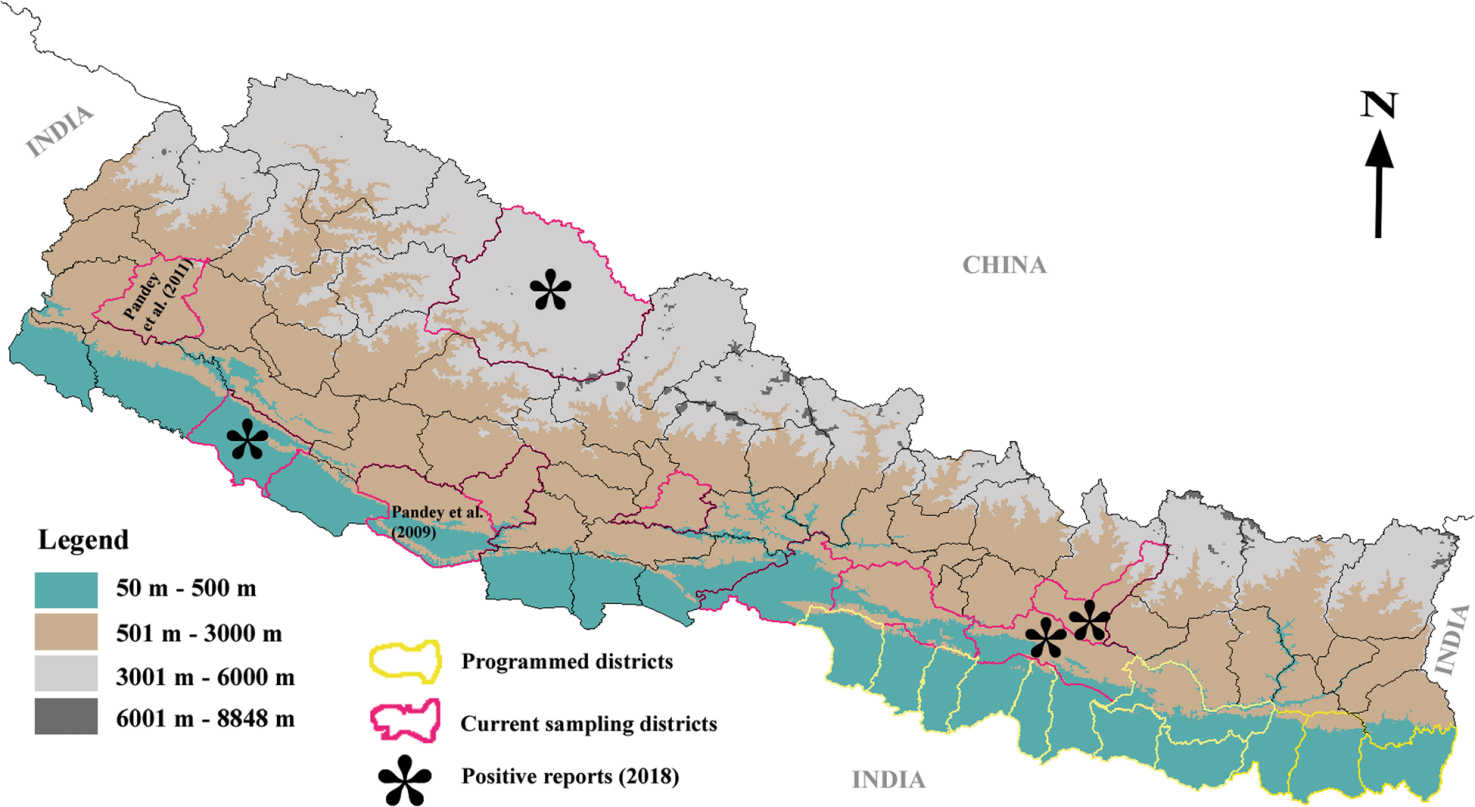
Map of Nepal showing the sampling districts (non-program districts). The map is prepared using Vector files available in DivaGIS webpage. (http://www.diva-gis.org)

Apart from the laboratory investigation, a standard questionnaire was also used to gather demographic information such as, gender, age, address and travel history. Blood samples (3–5 mL) were collected from the suspected VL patients (from different districts of Nepal: Fig. 2) at STIDH for routine diagnosis during October, 2015 to December, 2016. For PCR examination, the collected blood was spotted onto a Whatman–FTA classic card filter paper (GE Healthcare Ltd., Princeton, NJ) making 3–4 spots (approximately 25 μL/spot), ambient-dried and stored at 4 °C until processed.

### DNA extraction and nested-PCR amplification for *Leishmania* spp. identification

DNA was extracted from filter paper blood spots using QIAamp DNA Blood mini kit (Qiagen, Valencia, CA) following the manufacturer’s protocol. DNA was eluted to a final volume of 20 μL, and stored at − 20 °C until used for nested-PCR.

For molecular identification of the parasite, the variable regions of the kinetoplast minicircles DNA were amplified using *Leishmania* specific primer. The primers information and nested-PCR protocols have been detailed elsewhere [15]. Briefly, first round PCR was performed with 5 μL of template DNA and primers (CSB2XF – C/GA/GTA/GCAGAAAC/TCCCGTTCA and CSB1XR – ATTTTTCG/CGA/TTTT/CGCAGAACG), and the reaction condition was set as 94 °C for 2 min followed by 40 cycles of amplification (94 °C for 30 s, 54 °C for 1 min, and 72 °C for 1.5 min) and final extension at 72 °C for 10 min. In the second round PCR, 5 μl of first round diluted PCR product was used as template together with the following primers: 13Z – ACTGGGGGTTGGTGTAAAATAG and LiR – TCGCAGAACGCCCCT. The reaction condition was set as 94 °C for 2 min followed by 40 cycles of amplification (94 °C for 30 s, 56 °C for 1 min, and 72 °C for 40 s) and final extension at 72 °C for 10 min. The PCR products were resolved on 1.5% agarose gel, stained with ethidium bromide, and visualized under UV light. DNA from previously characterized culture isolates of *Leishmania* spp. were used as positive controls in all assays while PCR-grade water (Milli-Q) was used as negative control. The sizes of the first and second PCR product were approx. 750 bp and 720 bp respectively.

### Amplicon purification and sequencing

The PCR product was purified using ExoSAP-IT Express (Affymetrix, Inc., CA, USA) according to the manufacturer’s manual. For this, 5 μl of PCR product and 2 μl of Exosap was mixed and incubated at 37 °C for 4 min followed by 80 °C for 1 min. The purified product was subjected to sequencing reactions using BigDye™ Terminator v3.1 Cycle Sequencing Kit (Applied Biosystems, CA, USA) following the instructions provided by the manufacturer. These single stranded sequences were further purified by using Big Dye Xterminator™ Kit (Applied Biosystems). Finally, the purified amplicons were sequenced in an automated 3500XL Genetic Analyzer (Applied Biosystems).

The quality of raw sequences were checked through base calling in sequencer v. 4.1.4 (GeneCodes Corporation, MI, USA) and consensus sequences were generated using both strands. Subsequently, the final sequence obtained from each sample was subjected to Nucleotide Basic Local Alignment Search Tool (BLAST) to search similarity with the *Leishmania* spp. sequences deposited on National Center for Biotechnology Information (NCBI) database and identify the *Leishmania* spp.

### Data analysis

Data on VL confirmed cases (2008–2017) was retrieved from Annual Health Reports, Department of Health Services, Ministry of Health and Population, Government of Nepal. Data were analysed for disease trends by using non parametric Mann-Kendall trend tests in XLSTAT software. *P*-value < 0.05 was considered statistically significant (i.e. significantly decreasing or increasing VL trend).

### Ethical statement

This study was approved by the Ethical Committee of Nepal Health Research Council. Blood samples were collected after obtaining written informed consent from patients and the concerned hospital. In the case of minors, consent was given by parents or guardians.

## Results

**Fig. 3:**
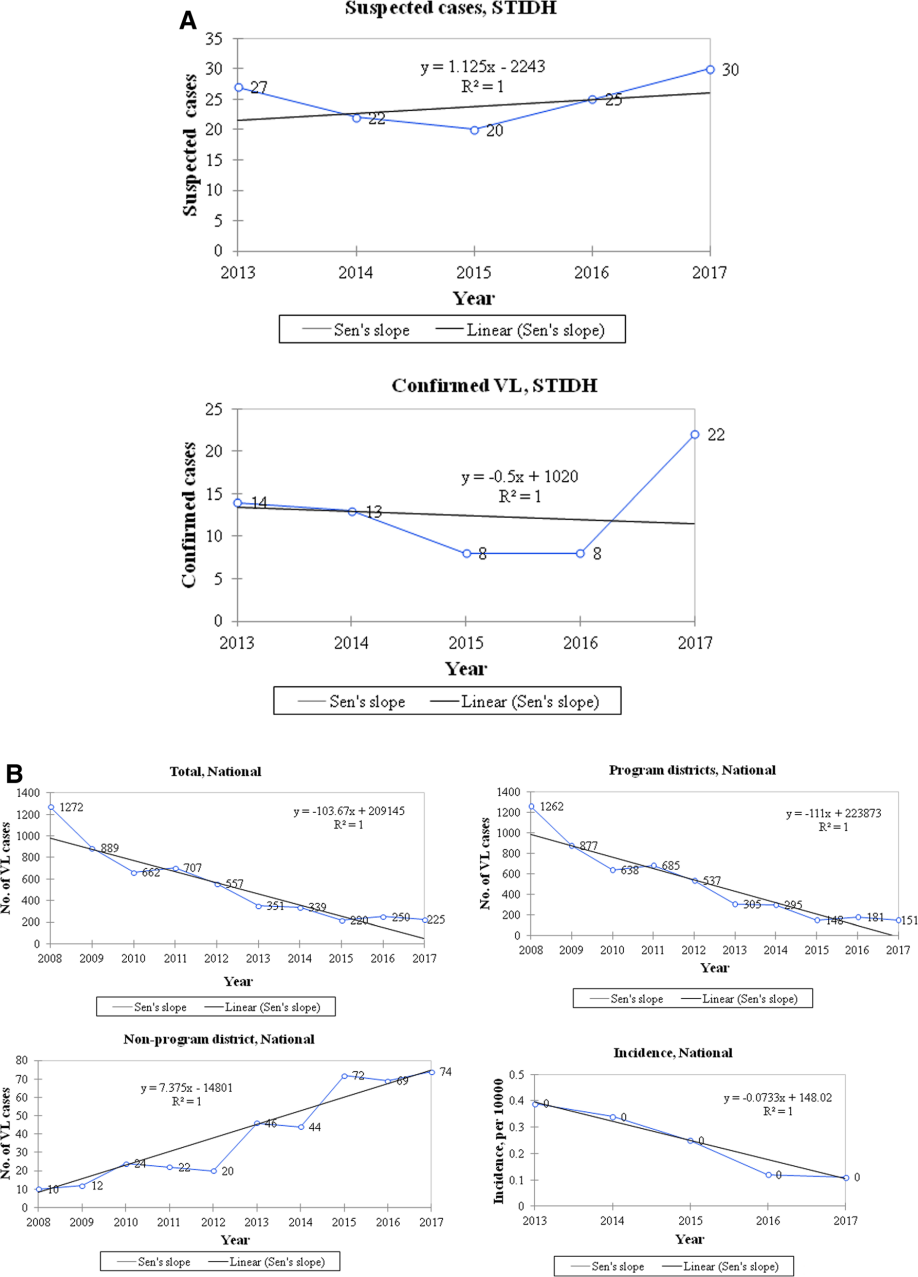
Recent trends of annual VL cases (2008–2017) reported in Nepal demonstrates the inclining situation in the non-program districts while the reverse in the program districts. **a**. Total number of annual VL cases admitted/treated in the central hospital (STIDH) from the non-program districts was from the present study. VL, Visceral leishmaniasis; STIDH, Sukraraj Tropical and Infectious Diseases Hospital. **b**. National data of annual VL cases in program and non-program districts, and overall incidence (limited to national data available for recent 10 years) were obtained from the annual reports of Ministry of Health, Department of Health Services, Government of Nepal (9). Non parametric Mann-Kendall trend tests were used to analyse the VL trends in Nepal over the last 10 years. Annual numbers of cases were plotted and a trend line with Sen’s slope has been presented along with its equation in the graph. Incidence has been expressed as per 10,000 populations at risk. *P*-value < 0.05 was considered statistically significant or reject null hypothesis that there is no monotonic trend (i.e. significantly decreasing or increasing VL trend)

To understand the trend of VL in past 10 years in Nepal, we plotted the number of VL cases from both program and non-program districts (national totals) including the cases from STIDH during 2013 to 2017 (Fig. 3a). The trends of VL cases during this period were clearly different between program and non-program districts of Nepal. In the past 10 years, there was significantly decreasing trend of VL cases in the program districts of Nepal (*p* = 0.001), while a significantly increasing trend of VL cases was observed in non-program districts (*p* = 0.002) (Fig. 3b). The national trend for overall incidence of VL also significantly decreased over this time period (*p* = 0.001). Although the number of cases attended at the STIDH is smaller than the national totals (2013–2017 and study year 2016), the trend observed in this hospital was also quite similar to that of the country’s national scenario. Furthermore, STIDH is the only central specialized hospital for treatment of tropical diseases including leishmaniasis in the country and these 14 cases represent all of the cases at the central hospital (STIDH) over the study period (2016). National reports consist of all the VL cases reported throughout the country (peripheral hospitals, health facilities) including the cases from STIDH.

Analysis of VL cases from STIDH showed that the age of suspected VL patients ranged from 10 to 70 years, with a median age 24, (47–23) years and all these cases were male (*n* = 14). Travel history indicated that none of patients had visited to program districts of Nepal and endemic areas of India.

**Fig. 4:**
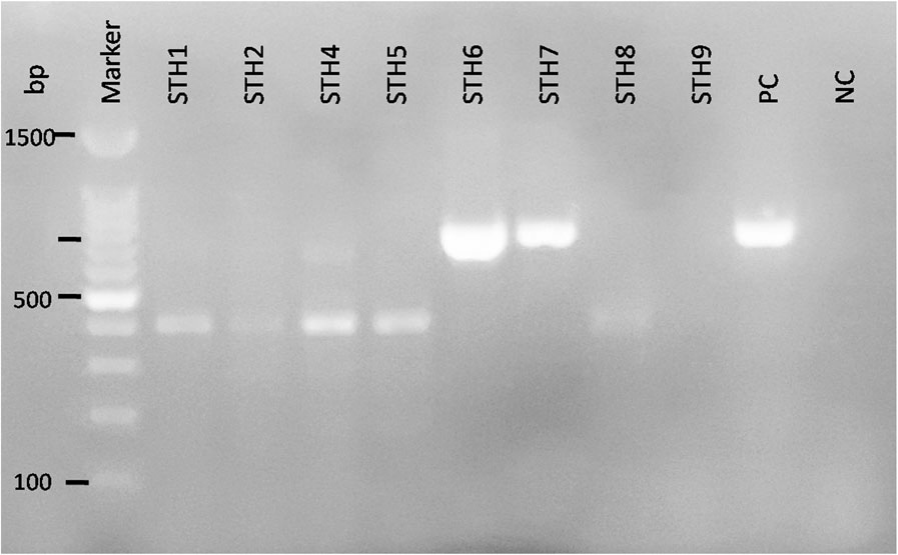
Molecular identification of VL cases from the non-program districts of Nepal. Agarose gel electrophoresis showed the sizes of amplified products by nested PCR using primers targeting kintetoplast minicircle DNA of *Leishmania* spp. Amplified band of the target size (720 bp) were also confirmed to be of *Leishmania* spp. by Sanger’s sequencing and BLAST. Samples 1 to 9 were collected from VL suspected patients visited to Sukraraj Tropical and Infectious Diseases Hospital (STIDH = STH) from different districts. Two different bands were detected while the band size (approx. 720 bp) is the positive cases for VL (STH6, STH7 and PC). PC for positive control and NC for negative control

Out of 14 patients, initial screening was performed by rK39 dipstick test and two patients were required bone marrow aspiration for confirmation (Fig. 1). Two bone marrow aspiration positive cases were also positive by PCR. Out of 14 VL suspected cases, six were positive by rK39, six were negative by rK39 and two cases remained inconclusive for rK39. Only 2 (33.3%) rK39 positive cases were positive by PCR. Sociodemographic, clinical and molecular results are presnted in Table 1. Of the 14 clinically suspected VL cases, 4 (28.6%) samples had the expected band size (approx. 720 bp); the same as the positive control (*L. donovani*) (Fig. 4). These PCR positive cases were from Chitwan, Dang, Dolpa and Sindhuli districts of Nepal (Fig. 2). A case of VL from Nepal’s mountain region (Dolpa) reported previously [11] was also included in the present study. PCR positive samples were sequenced to determine the identity of the PCR product. The sequences were deposited in GenBank (Accession number: MK803363–65). Using the nucleotide BLAST database search through the NCBI, we found that the PCR positive samples possessed high homology with the *L. donovani* complex, the causative agent of VL.

**Table 1:** Demographic profile of VL suspected patients

SN	Sample ID	Sex	Age	Address	Microscopic/RDT	PCR result
1	KCH1	M	10	Dolpa	BM (+)/rK39 (+/−)	Positive
2	TU1	M	70	Syanja	rK39 (−)	Negative
3	STIDH1	M	34	Dang	rK39 (+)	Negative
4	STIDH2	M	16	Ramechap	rK39 (−)	Negative
5	STIDH4	M	26	Makawanpur	rK39 (+)	Negative
6	STIDH5	M	21	Ramechap	rK39 (−)	Negative
7	STIDH6	M	49	Dang	rK39 (+)	Positive
8	STIDH7	M	60	Chitwan	BM (+)/rK39 (+/−)	Positive
9	STIDH8	M	23	Dang	rK39 (−)	Negative
10	STIDH9	M	47	Pyuthan	rK39 (+)	Negative
11	STIDH10	M	40	Syanja	rK39 (−)	Negative
12	STIDH12	M	44	Ramechap	rK39 (+)	Negative
13	STIDH15	M	23	Dang	rK39 (−)	Negative
14	STIDH16	M	48	Sindhuli	rK39 (+)	Positive

*VL* Visceral leishmaniasis, *M* Male, *RDT* Rapid diagnostic test, *PCR* Polymerase chain reaction, *BM* Bone marrow

Together, our data suggest that - 1) the number of VL cases was found to be increasing in non-program districts of Nepal despite the successful reduction in program districts, and 2) VL cases from these non-program districts were confirmed by a molecular approach (sequencing). It is high time to focus the VL control program in non-program districts to achieve the elimination goals.

## Discussion

All four districts (Chitwan, Dang, Sindhuli and Dolpa) with cases positive by molecular tools are located in the VL non-program districts. Chitwan and Dang districts are located in the lowland areas (250–2500 asl) of Nepal while Sindhuli is in the hilly region (300–3000 asl) and Dolpa is in the mountain region (1500–7500 asl) (Fig. 2). Chitwan district is located along the bank of Narayani river (one of the 3 largest rivers in the country) while Dang is located in the valley. Chitwan and Sindhuli are in an immediate geographical proximity to endemic districts, there will be higher chance to transmit the diseases in those districts due to the movement of people. Although the Government of Nepal, Ministry of Health and Population is committed to eliminate VL by 2020, the rise in incidence of cases from non-program districts could be a major obstacle in achieving the set target [12–14]. The serial reports of such incidences may be attributed to the possibility of spread from program districts or the presence of VL parasites in non-program districts, and it was largely overlooked in the past. The study on vector dynamics was conducted at two high altitude districts of Nepal [14] and found local VL transmission. But extensive vector and parasite infection studies are yet to be conducted so as to ascertain the presence of parasite in other non-endemic areas with reported VL cases.

Ten years data of VL patients from both program and non-program districts presented in Fig. 3b clearly demonstrates an increasing trend in non-program districts. Increasing developmental projects/improved connectivity and trade/tourism in recent years have posed high risk of VL through the introduction/transmission of vector and *Leshmania* infected individuals into the non-program districts. And, the outcome is the presence of VL in areas previously thought to be free. VL prevalence is strongly associated with poverty and access to health care facilities in developing countries including Nepal [16]. In Nepal’s rural settings, houses with damp earthen floors are common, an ideal breeding site for sandflies, however data on actual distribution of the vector is lacking. Awareness raising programs and government support reduced the overall VL cases in Nepal [10] though largely overlooked in non-program districts. Global rise in temperature could play a vital role in increased adaptability of the vectors which subsequently increases the habitat area for the vector borne diseases pathogens worldwide [17–20]. Rising socioeconomic status of local people and their enhanced accessibility to patient diagnosis and parasites detection could also have impacted this incidence. Previously inaccessible places or places without proper health facilities have now improved situation [19–21] both in private and government sectors. Nevertheless, the increasing case reports is a fact, though the reason behind it might not be explained by a single attribute instead it could be a cumulative outcome of multidimensional circumstances and needs to be properly investigated and addressed.

The VL control program has greatly benefited from rK39 test, however it is inadequate when it comes to elimination phase due to declining positive predictive values. Despite the claimed sensitivity and specificity [22], the rK39 test (antibody detection) has several shortcomings, such as need of expert clinical interpretation, false positive results due to post-recovery antibody retention for years and among residents of VL endemic areas [23], and time required for detectable antibody production delaying the diagnosis. To address this gap, a more specific test is required [24, 25] and PCR is a better alternative due to the following advantages: applicable to varieties of samples at early acute phase helping transmission reduction by timely diagnosis [24, 25], less invasive than bone-marrow aspiration [26, 27], prompt species identification (*L. donovani* complex) [26, 27], and improved sensitivity/ specificity using buffy coat [28], Although we did not compare the diagnostic performance in our setting due to small sample size, PCR is certainly useful in a country embarking elimination like Nepal where malaria, typhoid, tuberculosis, brucellosis and leptospirosis are co-endemic.

Our study provides important insights into the VL expansion to non-program districts which is strongly supported by use of PCR/sequence homology-based method in VL confirmation. However, due to the limited resources in our exploratory study, we could not collect larger number of samples and carry out necessary diagnostic tests for more conclusive results. Similarly, distribution of vector and parasites prevalence in all non-program districts was not studied. Not all the patients underwent bone marrow aspiration for diagnosis since the rK39 had discordant results with PCR for some patients. A small amount of blood was collected on filter paper and used for DNA extraction which might compromise the DNA concentration although we did not confirm it.

## Conclusion

With the rising number of VL cases reported from non-program districts of Nepal, it has become imperative for formulating new strategies that can overcome the present hassle of elimination obstacles as well as prevent an impending epidemic in low to high mountainous areas. Therefore, these findings should be considered as a signal for inception of a robust strategic plan for accommodation of non-program districts in the elimination program. Moreover, innovative strategies including molecular investigation should also be institutionalized to understand the origin and spread of these strains especially at this pre-elimination phase of VL.

## Abbreviations

BLAST: Basic Local Alignment Tool
DNA: Deoxyribonucleic acid
NCBI: National Center for Biotechnology Information
PCR: Polymerase chain reaction
STIDH: Sukraraj Tropical and Infectious Disease Hospital
VL: visceral leishmaniasis
